# Sudden Death from Spontaneous Coronary Artery Dissection due to Polyarteritis Nodosa

**DOI:** 10.7759/cureus.1737

**Published:** 2017-10-02

**Authors:** Cyrus M Munguti, Paul M Ndunda, Tabitha M Muutu

**Affiliations:** 1 Internal Medicine, University of Kansas School of Medicine - Wichita; 2 Pediatrics Observership, Wesley

**Keywords:** spontaneous coronary artery dissection, polyarteritis nodosa, vasculitis associated sudden cardiac death, sudden cardiac death

## Abstract

Spontaneous coronary artery dissection (SCAD) is an emerging and rare cause of acute coronary syndrome and sudden cardiac death. While it was previously reported among young females with fibromuscular dysplasia, new literature indicates that this condition could occur in older populations. Polyarteritis nodosa (PAN) causes systemic necrotizing vasculitis which typically affects small to medium-sized muscular arteries and could affect the coronary arteries. A few case reports of PAN causing acute coronary artery disease have been reported in the literature. We report a case of a 62-year-old lady who presented with abdominal pain and died abruptly during her stay; she was found to have PAN-associated SCAD on autopsy.

## Introduction

While atherosclerosis is the leading cause of coronary artery disease in adults, spontaneous coronary artery dissection (SCAD) has emerged as a rare cause of acute coronary syndrome (ACS) and sudden cardiac death [1-2]. Previously, SCAD was described among young females with no traditional coronary disease risk factors and especially in association with fibromuscular dysplasia. Advances in the understanding of this condition indicate a wide variety of associations and triggers. Arteriopathy from vasculitis causing weakness of the vasa vasorum has been described as a key factor in the pathophysiology of SCAD. The vasculitis associated with polyarteritis nodosa (PAN) affects small to medium-sized muscular arteries and rarely affects coronary arteries.

## Case presentation

A 62-year-old-female was admitted to the general surgery service with a one-day history of severe abdominal pain. She described the pain as sharp, constant, initially in the periumbilical region, and associated with nausea and vomiting. She endorsed subjective fever, chills, and a 20-pound unintentional weight loss over the preceding six months. The pain later progressed to the epigastric and upper quadrant regions and radiated to the back. There were no known exacerbating or relieving factors. Her past medical history was significant for hypertension, hyperlipidemia, and anomalous right coronary artery origin, for which she had undergone single vessel coronary artery bypass graft (CABG) surgery in 2007. Her home medications included amlodipine, atenolol, quinapril, furosemide, clonidine, and lovastatin. Her family history was significant for coronary artery disease in her father at the age of 50 years. She was married with five children and denied smoking, alcohol use, and illicit drug use.

On examination, she was alert and in distress due to pain. Her vital signs were normal. The abdomen was non-distended, soft and tender to palpation in the right upper quadrant and epigastric regions, with no rebound tenderness or guarding. Her cardiovascular exam was unremarkable with a regular rhythm, normal heart sounds, and no murmurs. Peripheral pulses were equal on all limbs, with no radio-femoral delay. The chest was clear on auscultation and limbs were warm without any edema. There were no focal signs on neurologic examination, and her skin was dry and intact with no lesions or rashes. Initial laboratory tests were significant for white blood cell count of 20.0 x 10^3^ per microliter. She had normal renal function and liver function panel including aspartate aminotransferase (AST), alanine aminotransferase (ALT), bilirubin, and albumin was normal. Urinalysis was negative for red blood cells (RBCs), protein, nitrites, and leucocyte esterase. A liver ultrasound showed a thickened gall bladder wall, and she underwent laparoscopic cholecystectomy on hospital Day 2 and reported subjective overall improvement by Day 3. On the fourth day of hospital stay, she complained of a headache and chills, but denied chest pain, cough, or shortness of breath. Her vital signs remained normal. The next day, she was found unresponsive with an asystole on the monitor and failed to respond after 30 minutes of resuscitation per standard advanced cardiac life support (ACLS) guidelines and was pronounced dead and an autopsy was done.

**Figure 1:**
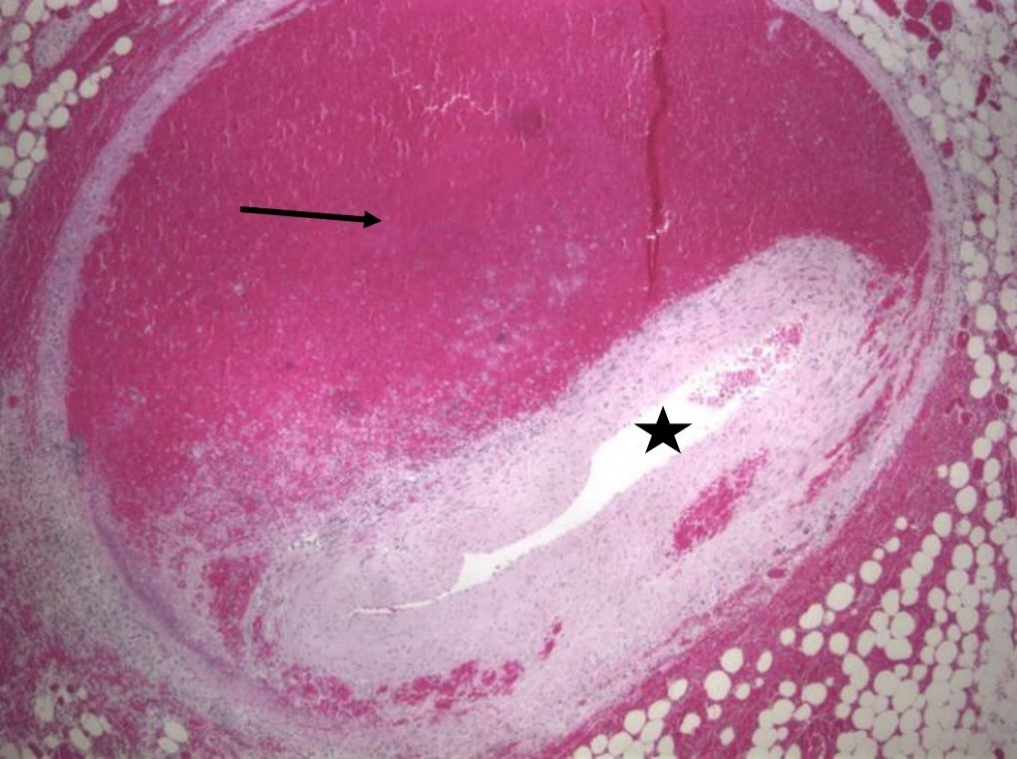
Cross-section of the posterior descending coronary artery with intramural hematoma (arrow) and luminal collapse (star)

**Figure 2:**
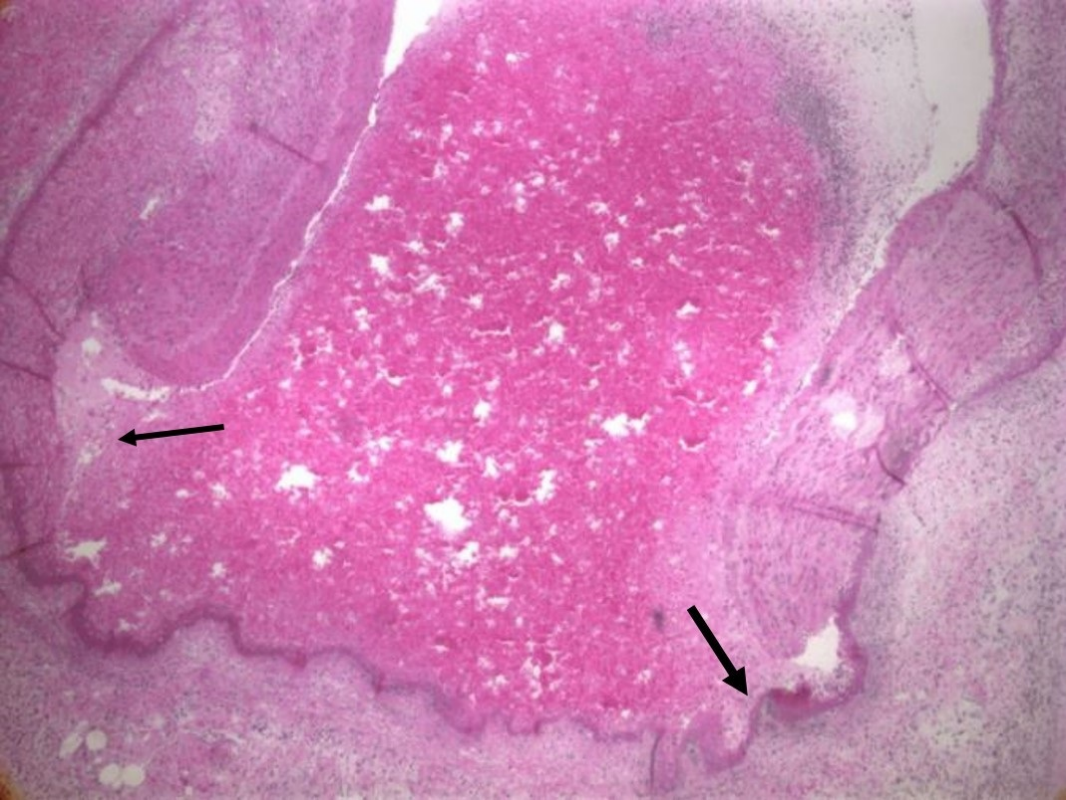
High-power view of a cross-section of the posterior descending coronary artery showing segmental fibrinoid necrosis associated with polyarteritis nodosa (arrows)

Grossly, the heart had a roughened pericardial surface on the right side compatible with the right internal mammary artery bypass graft site to the right coronary artery which was patent. The right coronary artery had an anomalous origin from the left coronary cusp and traversed between the pulmonary artery trunk and the aortic root. Serial sectioning the coronary arteries showed marked narrowing of the posterior descending artery. However, there was no significant atherosclerosis or thrombosis in the main coronary arteries and their branches. The tricuspid, pulmonic, mitral, and aortic valves were normal. There was no evidence of any old or new infarcts on myocardial sections. Microscopy of arterial sections revealed segmental fibrinoid necrosis (Figure 1 and Figure 2) with dissection with blood within the media leading to narrowing and occlusion of the posterior descending artery. There was no significant atherosclerosis or thrombosis in the main coronary arteries and their branches. She also had vasculitis and dissection of multiple vessels in the abdomen including the superior mesenteric artery, left colic artery, left gastric artery, pancreaticoduodenal artery, hepatic artery, and small artery branches in the kidneys. This segmental fibrinoid necrosis seen in multiple small and medium arteries was consistent with polyarteritis nodosa with some of the arteries such as the coronary artery branch having dissection of blood within the media leading to narrowing or occlusion of their lumens. The cause of death was suspected to be due to cardiac arrhythmias from posterior descending artery occlusion since myocardial sections did not show any evidence of new or old ischemic injury.

## Discussion

Spontaneous coronary artery dissection is an uncommon but under-recognized cause of ACS [1]. SCAD is the spontaneous separation of the coronary artery wall, which can occur in both atherosclerotic coronary artery disease and non-atherosclerotic coronary arteries, but the term SCAD is reserved for the non-atherosclerotic form [3]. Recent series using careful coronary angiography or advanced intracoronary imaging techniques suggest that the prevalence may be as high as one percent to four percent of all ACS cases [4-6]. It typically affects young women with few or no risk factors for atherosclerotic vascular disease. SCAD was associated with ACS in up to 42% of women under the age of 50 years in one series [5]. In one autopsy series [7], SCAD was reported in 0.5% of cases of sudden cardiac death, although this prevalence could be an underestimate.

The pathophysiology of SCAD is poorly understood, though two mechanisms have been described [3]. The intimal tear hypothesis suggests that a primary disruption in the intimal integrity on the luminal surface creates an entry point for blood, leading to the formation of intramural hematoma and the separation of the arterial wall. The medial hemorrhage hypothesis supposes that the primary event is the spontaneous rupture of a weakened vasa-vasorum leading to hemorrhage into the arterial wall. The presence of non-atherosclerotic arteriopathy compounded with a precipitating event often leads to the development of SCAD. Such arteriopathies include fibromuscular dysplasia, connective tissue diseases such as Marfan syndrome and Ehlers-Danlos syndrome, and systemic inflammatory diseases including systemic lupus erythematosus, polyarteritis nodosa, eosinophilic granulomatosis with polyangiitis (Churg-Strauss syndrome), inflammatory bowel disease, hormonal therapy, and coronary artery spasm. Activities reported to precipitate SCAD include Valsalva type activities, intense exercise, labor and delivery, intense emotional stress, and recreational drugs such as cocaine [8].

Several case reports and series on sudden death due to PAN have been published. Most of these cases were associated with coronary arteritis with or without myocardial infarction but no coronary dissection. Sudden death from SCAD due to previously undiagnosed PAN is very rare. Chu et al. [9] reported a case of a 51-year-old female with PAN that presented with a spontaneous left anterior descending (LAD) artery dissection and ST-segment elevation myocardial infarction that was initially treated medically with an intravenous tissue plasminogen activator. However, she needed rescue percutaneous coronary intervention (PCI) twice due to persistent chest pain and occurrence of new coronary dissections in the LAD and the left marginal artery. The diagnosis of vasculitis in that patient was suspected after the celiac, superior mesenteric, renal, and right axillary arteries revealed areas of aneurysmal dilatations and stenosis consistent with vasculitis on computed tomography (CT) angiography. The patient improved with steroid therapy and was asymptomatic by discharge and at one-month follow-up. Canpolat et al. [10] reported a case of a 23-year-old female with PAN for 15 years on prednisone and cyclophosphamide who presented with acute inferior non-ST elevation myocardial infarction. She was treated with PCI and appropriate medications for non-ST segment myocardial infarction. Interestingly, the erythrocyte sedimentation rate (ESR) and C-reactive protein levels for assessment of disease activity were normal, and therefore no additional immunosuppressive therapy was given. The patient had an uneventful hospital course and was asymptomatic at six months and one-year follow-up.

In retrospect, our patient presented with symptoms and signs that although nonspecific, should have raised suspicion of vasculitis. She had a history of fever and a 20-pound weight loss. Also, on a retrospective review of the abdominal CT scan with contrast, there was superior mesenteric dissection. The autopsy had no focal myocardial ischemic findings, although she had a posterior descending coronary artery dissection with occlusion and no other explanation for her sudden death. The cause of death was, therefore, most likely an arrhythmia and cardiac arrest from acute coronary dissection with luminal occlusion. Even though she had a history of a single vessel CABG surgery in her 40s, this was done for an anomalous origin of the right coronary and may or may not have predisposed her to SCAD.

## Conclusions

Our patient had dissections of multiple blood vessels in the abdomen including dissection of the posterior descending coronary artery. Histology revealed segmental fibrinoid necrosis in keeping with PAN. Her sudden death was most likely from arrythmias following the dissection and occlusion as there was no ischemic changes on myocardial cross-sections. Even though uncommon, coronary dissection should be considered in the differential diagnosis in patients with polyarteritis nodosa presenting with an acute coronary syndrome or sudden death.
